# Psychometric properties of the Hebrew version of brief mindful self-care scale: A translation and validation study

**DOI:** 10.1371/journal.pone.0330524

**Published:** 2025-08-21

**Authors:** Nasra Abdelhadi, Irit Bluvstein, Ronit Kigli-Shemesh, Semyon Melnikov

**Affiliations:** 1 Department of Nursing Sciences, Steyer School of Health Professions, Gray Faculty of Medical and Health Science, Tel Aviv University, Israel; 2 Herczeg Institute on Aging, Tel Aviv University, Israel; 3 Merhavim Mental Health Centre, Be’er Ya’akov, Israel; University of Maribor, SLOVENIA

## Abstract

**Background:**

Mindful self-care (MSC) integrates mindfulness into daily routines to enhance physical, mental, and emotional well-being. MSC is vital for nurses due to the high-stress nature of their work, which often leads to burnout and compassion fatigue. The Brief Mindful Self-Care Scale (BMSCS) was developed to measure MSC across six domains. However, there is limited research on MSC in Israeli nurses, and no Hebrew version of the BMSCS has been validated.

**Objectives:**

This study aimed to translate and validate the Hebrew version of the BMSCS and assess its psychometric properties among Israeli nurses.

**Methods:**

A cross-sectional study was conducted with 845 nurses recruited via a convenience sample. The BMSCS was translated into Hebrew using forward and backward translation. Psychometric analyses included exploratory and confirmatory factor analyses (EFA and CFA), item discrimination, and internal consistency (Cronbach’s alpha). Data were analysed using R software.

**Results:**

EFA using maximum likelihood estimation with Promax rotation revealed a six-factor solution accounting for 49% of the total variance. CFA results confirmed the model fit after excluding two low-loading items (PC2 and PC6), with goodness-of-fit indices meeting predefined criteria (e.g., RMSEA = 0.049, CFI = 0.949). The Cronbach’s alpha for the total scale was 0.87, with subscale reliability ranging from 0.70 to 0.89. Subscale correlations supported the internal structure.

**Conclusions:**

The Hebrew BMSCS demonstrated strong psychometric properties, providing a reliable and structurally sound tool for assessing MSC in Israeli nurses. This instrument can guide interventions to enhance nurses’ well-being and inform future research in high-stress healthcare settings.

## Introduction

Self-care is a proactive process involving daily activities that individuals engage in to maintain and enhance their physical, mental, and emotional health [1]. Among the self-care definitions is “a process of maintaining health through health-promoting practices and managing illness” [2], often conceptualized through frameworks such as Orem’s Self-Care Deficit Nursing Theory [3], or the Self-Care Theory of Heart Failure [4]. Beyond physical wellness, self-care also encompasses mental health, emphasizing its holistic role in promoting overall well-being [5]. In this context, mindfulness—rooted in Buddhist contemplative traditions — offers a powerful approach to enhancing mental and emotional self-care by encouraging individuals to pay attention to their present moment experience with openness, curiosity, and without judgment [6]. Mindful self-care (MSC) incorporates mindfulness principles into everyday self-care practices, creating a dynamic process that emphasizes awareness, intentionality, and non-reactivity in promoting health and well-being. MSC involves integrating mindfulness into daily routines through both formal activities, such as yoga or other mind-body practices, and informal practices, such as performing everyday tasks with heightened awareness [7]. The psychological rationale behind MSC is that when individuals engage in self-care mindfully, they are more likely to notice early signs of distress, respond to their needs with compassion, and avoid burnout [8]. Mindful self-care is particularly relevant in high-stress professions like nursing, where compassion fatigue and burnout are common challenges. Mindful self-care has been associated with self-compassion and resilience among palliative care providers [9]. By cultivating mindful self-care, nurses can enhance their professional well-being and deliver higher-quality patient care [10, 11].

The Mindful Self-Care Scale (MSCS), developed by Cook-Cottone and Guyker (2017), provides a structured tool to assess how individuals perceive their engagement in mindful self-care across physical, cognitive, emotional, and social domains [7]. This scale incorporates six subscales to address needs and demands in a way that supports well-being and personal effectiveness: physical care and relaxation, supportive structure, supportive relationships, mindful awareness, self-compassion and purpose, and mindful relaxation. The MSCS allows individuals to identify areas of strength and weakness in their self-care practices, enabling the development of targeted improvement strategies.

### Mindful self-care in health care professionals

Interacting with patients who have severe or critical illnesses can negatively impact on the emotional and physical health of healthcare professionals. A previous study demonstrated that secondary traumatic stress, compassion fatigue, and burnout among healthcare professionals working in adult and pediatric intensive care units, as well as in departments treating patients with serious illnesses, affected all hospital staff regardless of profession [12]. At the same time, MSC was positively associated with well-being in medical residents and negatively with distress and depression. In the long run, mindful-based self-care protects medical residents against depression caused by burnout and stress [13]. Similarly, positive relationships were found between MSC and professional quality of life and meaning made among palliative care workers [14]. Likewise, a controlled trial among nurses demonstrated that a brief mindful self-care intervention led to significant reductions in burnout and depressed mood, with these improvements persisting at 6 months for the intervention group [15].

Although the Brief Mindful Self-Care Scale (BMSCS) has been validated in other languages and populations, its psychometric properties have not been examined in Hebrew or within the Israeli cultural context. Given that translation can affect semantic and conceptual meaning, and that self-care practices are influenced by cultural and occupational factors—particularly among healthcare professionals—it is essential to validate the Hebrew version. This study aims to establish the internal consistensy and factorial structure of the BMSCS in a sample of Israeli nurses, thereby enabling its use in local clinical practice and cross-cultural research.

## Methods

### Participants

The study used a cross-sectional research design and adhered to the Strengthening the Reporting of Observational Studies in Epidemiology (STROBE) guidelines [16]. Data was collected through an online questionnaire administered to nurses using the web-based survey platform Qualtrics (Provo, UT, USA). The sample size was determined based on established guidelines, which recommend a minimum of 100 participants for EFA [17] and at least 150 participants for CFA [18]. A total of 845 nurses participated in the study, recruited via convenience sampling. Of the 845 nurses who initially participated, 112 responses were excluded from the psychometric analyses due to incomplete or inconsistent data (e.g., partial survey completion or missing responses to BMSCS items). Therefore, the final analytic sample included 733 complete cases used for EFA, CFA, and reliability analyses.

### Data collection

Data was collected from March 12 to June 27, 2023. The questionnaire was distributed by 28 registered nurses who were students in a master’s degree nursing program at Tel Aviv University. As part of their clinical research course, each student was assigned to distribute the questionnaire to a convenience sample of 30 registered nurses. The respondents were primarily nurses who were friends or colleagues of the students. Questionnaires were distributed using the Qualtrics software platform, guaranteeing a secure and confidential data collection procedure. The study employed a self-administered questionnaire in Hebrew comprising closed-ended questions. Before accessing the online questionnaire, participants were provided with a detailed explanation of the research objectives and their rights, followed by collecting their informed consent.

Furthermore, it was explained that the questionnaire was anonymous, with no identifying information, and that the findings would only be used for research. Participants were informed that they could discontinue filling out the questionnaire anytime. However, if they chose to complete it, they were required to answer all questions to ensure no missing data. A convenience sample of 10 nurses was selected to conduct a preliminary survey and invited to evaluate each item’s layout design and clarity. No further changes were required. To prevent duplicate responses, the Qualtrics platform was configured to allow only one submission per IP address.

### Scale translation

Professor Cook-Cottone granted permission for the translation work. First, BMSCS was translated into Hebrew by two authors, one RN with a PhD and an RN with an MA majoring in English. Then, two other authors, an RN and a psychologist, both with PhD, who were majoring in English, did the reverse translation. In a joint meeting between the four authors, an agreement was reached on the final version of the translation (S1 Appendix).

### Measures

#### Brief-mindful self-care (BMSC) scale.

Mindful self-care levels were assessed using the Brief Mindful Self-Care Scale (BMSCS), based on the original scale of 33 items developed by Cook-Cotton and Guyker [7] and subsequently shortened to 24 items by Hotchkiss and Cook-Cottone [19]. The scale is measured on a Likert scale from one to five, corresponding to never (1), rarely (2), sometimes (3), often (4), and regularly (5). The scale contains six domains: mindful relaxation, physical care, self-compassion and purpose, supportive relationships, supportive structure, and mindful awareness. Each subscale score is calculated by averaging the items within that subscale. The total score is calculated by summing all 24 item scores, resulting in a total score ranging from 24 to 120. The higher the total score, the higher the level of MSC.

### Data analyses

#### Items analysis.

To assess item discrimination, we used the critical ratio method, a classical approach based on comparing responses between high and low scoring participants [20]. Specifically, we divided the sample into the top 27% and bottom 27% based on total BMSCS scores and conducted independent samples t-tests to compare item scores between the two groups. This analysis evaluates whether each item can effectively distinguish between individuals with higher versus lower levels of mindful self-care.

### Reliability

The reliability of the translated BMSCS was evaluated using Cronbach’s alpha, with values of 0.70 or higher indicating acceptable internal reliability [21].

### Psychometric evaluation

#### Face Validity.

To assess quantitative face validity, a pilot study was conducted with 10 nurse participants. They were asked to provide feedback on the content, language, clarity, and simplicity of the translated BMSCS items.

### Factorial structure

Exploratory factor analysis (EFA) was performed on the translated BMSCS to examine its underlying factor structure and internal consistency. The analysis included 24 items grouped into six subscales. Relevant variables were extracted, and the correlation matrix was computed. Factors were extracted using maximum likelihood (ML) estimation, with Promax (oblique) rotation. The number of factors was set to six based on theoretical considerations. The adequacy of the data for factor analysis was assessed using the Kaiser-Meyer-Olkin (KMO) test and Bartlett’s Test of Sphericity. A KMO value above 0.6 indicated sampling adequacy, while a significant result for Bartlett’s test (p < 0.05) confirmed the interrelatedness of variables [22]. Factor loadings, eigenvalues, and the percentage of variance explained were examined to evaluate the scale’s dimensionality. Following the initial analysis, items PC2 and PC6 (the numbers of all items in the manuscript correspond to the numbers in the original scale of 33 items by Cook-Cotton and Guyker [7]) were excluded due to low factor loadings and the EFA was repeated to refine the factor structure. Eigenvalues were used to determine the number of factors to retain, following Kaiser’s criterion (eigenvalues > 1). A scree plot was generated to visually inspect the eigenvalues and identify the optimal number of factors based on the “elbow” point.

### Confirmatory factor analysis

Confirmatory factor analysis (CFA) was performed to evaluate the factor structure of the measurement model for the BMSCS. Two models were tested: the initial model (Model 1) included all items hypothesized to load on six latent constructs—Physical Care (PC), Supportive Relationships (SR), Mindful Awareness (MA), Self-Compassion and Purpose (SCP), Mindful Relaxation (MR), and Supportive Structure (SS). The revised model (Model 2) excluded items PC2 and PC6 based on low factor loadings. The fit of each model was assessed using several goodness-of-fit indices, including the relative Chi-square (χ²/df ratio) between 2 and 3, indicating acceptable fit; Root Mean Square Error of Approximation (RMSEA) values less than 0.05, indicating good fit; Comparative Fit Index (CFI) values greater than 0.95, indicating good fit; Tucker-Lewis Index (TLI) values greater than 0.95, indicating good fit; and Standardized Root Mean Residual (SRMR) values less than or equal to 0.05, indicating good fit [22].

### Statistical analyses

Data analysis was conducted using R software (version 4.4.2) and Analysis of Moment Structures (AMOS) version 30.0. Data manipulation, including cleaning and renaming variables, was performed using the dplyr package. The psych package facilitated psychometric calculations, including the computation of subscale means and total scores, EFA, psychometric analyses, and the creation of a scree plot. The Hmisc package was used to compute Pearson correlation matrices. CFA and visualization of the CFA models were performed using AMOS. The Standardized Root Mean Square Residual (SRMR) value was calculated separately using the fitMeasures() function from the lavaan package in R.

### Ethical considerations

The study was approved by Tel Aviv University’s ethics committee (approval number 0006284−2). Informed consent was obtained by requiring participants to click the “I agree” button at the start of the electronic questionnaire, which was mandatory to proceed.

## Results

### Descriptive statistics

This study included 845 nurses: 162 males (19%) and 679 females (80%), with an average age of 38 years (SD = 11). The majority (72%) were Jews and 24% were Arabs. More than three-fourths (78%) of the participants had a steady partner. Over half (59%) of participants had an RN BA/BSn education. The majority (72%) worked in hospitals. The average seniority as nurses was 11 years. More than half (61%) of participants perceived their health as good. Additional sociodemographic and professional information are shown in Table 1.

**Table 1:** Participants’ socio-demographic and professional characteristics (n = 845).

Variables	N (%)
Gender	
Male	162 (19.17)
Female Other	679 (80.36) 2 (0.24)
Missing	2 (0.24)
Ethnicity	
Jew	608 (71.95)
Arab	200 (23.67)
Other	27 (3.20)
Missing	10 (1.18)
Marital status	
Yes steady partner	657 (77.75)
No steady partner	183 (21.66)
Missing	5 (0.59)
Education	
RN	88 (10.41)
RN BA/BSn	498 (58.93)
RN MA/PhD	249 (29.47)
Missing	10 (1.18)
Shifts work	
Yes	628 (74.32)
No	216 (25.56)
Missing	1 (0.12)
Work Place	
Community clinics	141 (16.69)
Hospital	612 (72.43)
Administration/Management	14 (1.66)
Other	15 (1.78)
Missing	63 (7.46)
Smoking	
Yes	173 (20.47)
No	665 (78.70)
Missing	7 (0.83)
Health perception	
Bad	1 (0.12)
Not so good	21 (2.49)
Average	134 (15.86)
Good	517 (61.18)
Perfect	172 (20.36)
Age (years) (mean [SD])	37.71 (10.61)
Seniority (years) (mean [SD])	11.35 (10.69)

### Item analyses

The results demonstrated that all items significantly differentiated between groups (p < 0.001), with critical ratio values ranging from 7.78 to 19.57, indicating strong discriminative power across the scale. All items demonstrated adequate discrimination, as all critical values exceeded the threshold of 3.00. The item-total correlation (ITC) values (one factor/dimension) for all items, except for one (PC6, ITC = 0.10), were satisfactory, ranging from 0.22 to 0.73, indicating that each item was moderately correlated with the scale. The “Cronbach’s alpha if item deleted” values ranged from 0.70 to 0.86 (Table 2), indicating that removing any single item would not substantially improve the overall internal consistency of the scale, which remained high at 0.87.

**Table 2:** Item analysis for Hebrew version of the Brief Mindful Self-Care Scale (BMSCS) (N = 733).

	N	BMSCS items	M ± SD	Md	Critical ratio	Item total correlations	Cronbach’s Alpha if item deleted
Physical Care (PC)*							
1.	841	I ate a variety of nutritious foods (PC2)	3.68 ± 1.07	4.0	15.31	0.58	0.79
2.	838	I exercised at least 30–60 minutes (PC4)	2.70 ± 1.30	3.0	9.24	0.55	0.76
3.	838	I took part in sports, dance, or other scheduled physical activities (PC5)	2.46 ± 1.33	2.0	19.26	0.53	0.77
4.	838	I did sedentary activities instead of exercising (PC6)	2.77 ± 1.10	3.0	17.49	0.10	0.84
5.	835	I practiced yoga or another mind-body practice (PC8)	1.74 ± 1.11	1.0	12.75	0.37	0.81
Supportive Relationships (SR)							
6.	841	I spent time with people who are good to me (SR1)	3.74 ± 0.93	4.0	18.45	0.57	0.83
7.	832	I felt supported by people in my life (SR2)	3.90 ± 0.87	4.0	18.72	0.60	0.80
8.	837	I felt that I had someone who would listen to me if I became upset (SR3)	3.90 ± 0.94	4.0	19.38	0.58	0.80
9.	835	I felt confident that people in my life would respect my choice if I said “no” (SR4)	3.69 ± 0.92	4.0	19.46	0.59	0.84
Mindful Awareness (MA)							
10.	841	I had a calm awareness of my thoughts (MA1)	3.51 ± 0.88	4.0	11.51	0.72	0.83
11.	838	I had a calm awareness of my feelings (MA2)	3.51 ± 0.87	4.0	8.84	0.73	0.82
12.	836	I had a calm awareness of my body (MA3)	3.42 ± 0.91	3.0	10.26	0.71	0.86
Self-Compassion and Purpose (SCP)							
13.	842	I kindly acknowledged my own challenges and difficulties (SC1)	3.78 ± 0.80	4.0	17.07	0.41	0.82
14.	841	I engaged in supportive and comforting self-talk (SC2)	3.43 ± 1.05	4.0	13.73	0.44	0.78
15.	839	I gave myself permission to feel my feelings (SC4)	3.48 ± 0.98	4.0	16.92	0.36	0.81
16.	836	I experienced meaning and/or a larger purpose in my work/school life (SC5)	3.53 ± 0.91	4.0	14.87	0.40	0.81
Mindful Relaxation (MR)							
17.	835	I did something creative to relax (MR3)	2.51 ± 1.18	2.0	19.57	0.31	0.71
18.	838	I listened to relax (music, podcast, radio show, rainforest sounds) (MR4)	3.44 ± 1.10	4.0	12.13	0.30	0.70
19.	837	I sought out images to relax (art, film, window shopping, nature) (MR5)	3.33 ± 1.05	3.0	18.45	0.24	0.72
20.	829	I sought out smells to relax (lotions, nature, candles, baking) (MR6)	2.34 ± 1.21	2.0	18.56	0.22	0.73
Supportive Structure (SS)							
21.	839	I kept my work area organized to support my work tasks (SS1)	3.65 ± 0.99	4.0	10.94	0.29	0.80
22.	837	I maintained a manageable schedule (SS2)	3.65 ± 0.94	4.0	7.78	0.45	0.72
23.	833	I maintained balance between the demands of others and what is important to me (SS3)	3.47 ± 0.90	4.0	12.15	0.50	0.75
24.	834	I maintained a comforting and pleasing living environment (SS4)	3.64 ± 0.87	4.0	11.74	0.49	0.77

*In parentheses are the numbers of items in the original scale of 33 items by by Cook-Cotton and Guyker (Cook‑Cottone & Guyker, 2017).

### Factorial structure

**Fig 1:**
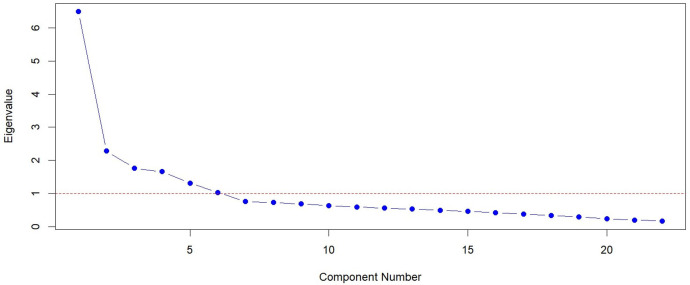
Scree plot for the translated version of BMSCS (22 items). Eigenvalues > 1.

Exploratory Factor Analysis (EFA) was initially conducted on all 24 items of the translated BMSCS. Two items from the Physical Care subscale—PC2 (“I ate a variety of nutritious foods”) and PC6 (“I did sedentary activities instead of exercising”)—were excluded due to low factor loadings of 0.38 and 0.25, respectively. Following their removal, EFA was re-run on the remaining 22 items. The factor loadings of the final 22-item Hebrew version of the BMSCS are presented in Table 3. The Kaiser-Meyer-Olkin (KMO) measure of sampling adequacy was 0.91, and Bartlett’s test of sphericity was significant (χ² = 11,330.6, df = 528, p < 0.001), supporting the suitability of the data for factor analysis. The items associated with the PC construct had factor loadings ranging from 0.47 to 0.96, explaining 10% of the total variance, with an eigenvalue of 3.22. The items associated with the SR construct had factor loadings ranging from 0.58 to 0.94, explaining 10% of the total variance, with an eigenvalue of 3.29. The items associated with the MA construct had factor loadings ranging from 0.75 to 0.98, explaining 8% of the total variance, with an eigenvalue of 2.76. The items associated with the SCP construct had factor loadings ranging from 0.51 to 0.85, explaining 8% of the total variance, with an eigenvalue of 2.78. The items associated with the MR construct had factor loadings ranging from 0.58 to 0.62, explaining 6% of the total variance, with an eigenvalue of 2.23. The items associated with the SS construct had factor loadings ranging from 0.60 to 0.90, explaining 7% of the total variance, with an eigenvalue of 2.13. As a result, 22 items were retained after reducing the number of variables, explaining a total cumulative variance of 49%. The six-factor structure, aligned with the original scale, was further supported by the scree plot, which showed a marked decline in eigenvalues leveling off after the sixth point (Fig 1).

**Table 3:** Brief Mindful Self-Care Scale (BMSCS): estimated factor loadings across the six factors (N = 733).

	MSCS items	F1	F2	F3	F4	F5	F6
*Physical Care (PC)*							
1.	PC4	0.96					
2.	PC5	0.87					
3.	PC8	0.47					
*Supportive relationships (SR)*							
4.	SR1		0.76				
5.	SR2		0.92				
6.	SR3		0.94				
7.	SR4		0.58				
*Mindful awareness (MA)*							
8.	MA1			0.98			
9.	MA2			0.96			
10.	MA3			0.75			
*Self-compassion and purpose (SCP)*							
11.	SC1				0.54		
12.	SC2				0.85		
13.	SC4				0.62		
14.	SC5				0.51		
*Mindful relaxation (MR)*							
15.	MR3					0.58	
16.	MR4					0.62	
17.	MR5					0.59	
18.	MR6					0.58	
*Supportive structure (SS)*							
19.	SS1						0.75
20.	SS2						0.90
21.	SS3						0.72
22.	SS4						0.60
	N. of items	5	4	3	4	4	4

### Confirmatory factor analysis (CFA)

**Fig 2:**
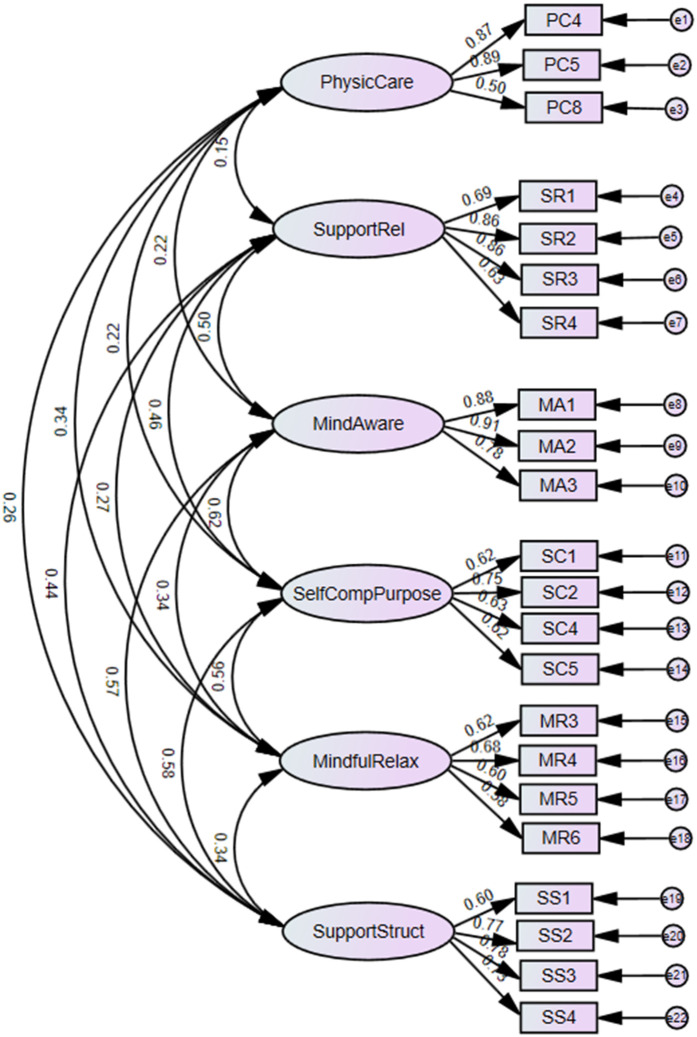
CFA measurement model 2 for the BMSCS. Physical Care (PC), Supportive Relationships (SR), Mindful Awareness (MA), Self-Compassion and Purpose (SC), Mindful Relaxation (MR), and Supportive Structure (SS). The numbers inside the rectangles indicate the statement numbers as they appear in the original 33-item scale by Cook-Cottone and Guyker (2017).

The CFA determines whether the latent variables, including physical care, supportive relationships, mindful awareness, self-compassion and purpose, mindful relaxation, and supportive structure, adequately fit the data. Twenty-two items were measured to evaluate the factor structure of the BMSC scale and its subscales. Fig 2 illustrates the factors’ loadings of the translated BMSC scale items. PC items loading range was between 0.50 and 0.89, MR items loading range was between 0.58 and 0.68, SCP items loading range was between 0.62 and 0.75, SR items loading range was between 0.63 and 0.86, MA items loading range was between 0.78 and 0.91, and SS items loading range was between 0.60 and 0.78. Table 4 presents the fit indexes for measurement Model 1, containing the original version of the scale with 24 items, and Model 2 includes 22 items after the exclusion of PC2 and PC6 items. Model 2 met the predefined criteria for acceptable goodness-of-fit. The relative chi-square is 3.016, indicating an acceptable but not excellent model fit. The RMSEA is 0.049, and the SRMR is 0.044, both indicating a good fit. Additionally, the CFI is 0.949, and the TLI is 0.933, both indicating an acceptable model fit.

**Table 4:** Model fit statistics statistics stepwise through the model revision (N = 733).

Model	Description	Chi-square	df	Chi-square/df	RMSEA	SRMR	CFI	TLI
1	Step 1: CFA of brief MSCS model 6 factors, 24 items	781.683	237	3.298	0.052	0.063	0.932	0.914
2	Step 2: 2 items excluded: PC2 and PC6, 22 items	585.110	194	3.016	0.049	0.044	0.949	0.933
Acceptable fit				<3	≤0.08	≤0.08	≥0.90	≥0.90
Good fit				<2	≤0.05	≤0.05	≥0.95	≥0.95

RMSEA=Root Mean Square Error of Approximation; SRMR= Standardized Root Mean Residual; CFI= Comparative Fit Index; TLI= Tucker-Lewis Index.

### Internal consistency

The internal consistency, measured using Cronbach’s Alpha, was calculated as 0.87, providing satisfactory evidence of the reliability of the BMSCS. Additionally, Cronbach’s Alpha values for the subscales were as follows: 0.79 for Physical Care, 0.84 for Supportive Relationships, 0.89 for Mindful Awareness, 0.74 for Self-Compassion and Purpose, 0.70 for Mindful Relaxation, and 0.82 for Supportive Structure.

### Subscale correlations of the B-MSCS

Table 5 presents the correlations among the indicators of the exogenous construct BMSCS (PC, SR, MA, SCP, MR, and SS) to examine the internal structure and interrelatedness of the subscales. Furthermore, Table 5 presents Cronbach’s alpha coefficients for the total scale and each subscale of the B-MSCS, calculated to evaluate reliability and internal consistency, with values ranging from 0.70 to 0.89. These Cronbach’s alpha values support the reliability and internal consistency of the total scale and all BMSCS subscales.

**Table 5:** BMSCS* subscales Cronbach’s alpha, and Pearson’s correlations among BMSCS subscales (N = 733).

Measure	PC	SR	MA	SCP	MR	SS
BMSCS	0.61**	0.76**	0.77**	0.49**	0.33**	0.44**
PC	0.79					
SR	0.18**	0.84				
MA	0.15**	0.45**	0.89			
SCP	0.22**	0.35**	0.48**	0.74		
MR	0.29**	0.18**	0.24**	0.42**	0.70	
SS	0.11**	0.37**	0.46**	0.41**	0.24**	0.82

*BMSCS = Brief Mindful Self Care Scale (includes the following subscales: PC = Physical Care; SR = Supportive Relationships; MA = Mindful Awareness; SCP = Self-Compassion and Purpose; MR = Mindful Relaxation; SS = Supportive Structure), α = Cronbach’s alpha.

Note: Values below the diagonal represent Pearson correlation coefficients; values on the diagonal are Cronbach’s alpha coefficients for each subscale. The Cronbach’s alpha for the total BMSCS scale was 0.87.

** p < 0.01.

## Discussion

The current study aimed to translate the BMSCS and evaluate its psychometric properties among the Israeli nurse population. The six original factors explained 49% of the variance. According to the presented findings, nurses around Israel utilized the MSC to identify its’ domains: mindful relaxation, physical care, self-compassion and purpose, supportive relationships, supportive structure, and mindful awareness. In terms of the instrument’s reliability, the obtained results demonstrate very satisfactory indexes, particularly after comparing the results to the original instrument’s validation, thus supporting the hypothesized factor structure of the translated scale. While the current study provides preliminary support for the factorial structure and internal consistency of the Hebrew version of the BMSCS, it is important to note that two items (PC2 and PC6) were removed during the confirmatory factor analysis to achieve acceptable model fit. PC2 (“I ate a variety of nutritious foods”) and PC6 (“I did sedentary activities instead of exercising”) had low factor loadings (0.38 and 0.25, respectively). From a theoretical perspective, PC2 reflects nutritional variety, which may be culturally interpreted in diverse ways and perceived as less central to mindful self-care than behaviors such as hydration or rest. PC6, a reverse-worded item, may have posed interpretation challenges, especially in translation, and focused on sedentary behavior rather than a direct mindful self-care action. Reverse-worded items are also known to sometimes perform poorly due to cognitive complexity [23]. Removing these items improved model fit and clarified the construct within the Hebrew-speaking nursing context.

As such, the findings do not fully confirm the original factorial structure of the BMSCS as validated in previous studies. Rather, the results suggest that a modified six-factor structure—closely aligned with the original—fits the data well in the current Israeli nurse sample. This partial replication may reflect cultural or linguistic differences and highlights the importance of further validation in additional samples. Our findings should therefore be viewed as supporting the psychometric robustness of a slightly adapted version of the BMSCS, while also pointing to the need for continued cross-cultural validation work.

The BMSCS model appears to align well with the emerging theoretical framework of nurses’ wellness and offers preliminary support for its internal structure and relevance in this population (Table 4). It emphasizes six key aspects of mindful self-care. In addition to providing good conceptual coverage, it eliminates low-loading or weakly contributing items. The Brief version of the translated MSC scale maintained internal consistency and reliability.

The Cronbach’s alpha of the Brief Mindful Self-Care Scale (MSCS), Hebrew version, presented a 0.87 value for the whole instrument. The results are like those of the original Brief Mindful Self-Care Scale (MSCS) [7], which had a Cronbach’s alpha of 0.89 for the total 33-item MSC Scale. The Cronbach’s alpha values for the 24-item version were comparable between the two versions of the scale—the original British [19] and the translated Hebrew version, respectively: Physical Care (0.77, 0.79), Supportive Relationships (0.77, 0.84), Mindful Awareness (0.86, 0.89), Self-Compassion and Purpose (0.78, 0.74), Mindful Relaxation (0.74, 0.70), and Supportive Structure (0.79, 0.82).

This study employed rigorous CFA methodology, considering both psychometric and conceptual criteria. The CFA results of the existing 24-item BMSCS, as reported by Hotchkiss and Cook-Cottone (2019), were comparable to the current findings, indicating acceptable goodness-of-fit for the Hebrew-translated BMSCS.

Our results were compared with previous studies that examined the psychometric properties of the MSCS/BMSCS. One study conducted in Brazil aimed to examine the psychometric properties of the Brazilian version of the MSCS in a sample of 336 palliative care providers [24]. Another study examined the psychometric properties of the Chinese version of BMSCS in a sample of 510 hospice nurses [25]. Similarly to the current study, both studies found that the CFA for the MSCS [24] and the BMSCS [25] supported the six-factor model. The Chinese BMSCS demonstrated criterion validity, with six factors loading on an eigenvalue greater than one [25].

Having established the translated BMSCS’s adequate psychometric properties, we concluded that it is well-suited for exploring MSC among nurses, given the inherently stressful nature of their work. While the current study provides evidence supporting the factorial structure and internal consistency of the Hebrew version of the Brief Mindful Self-Care Scale (BMSCS), it is important to acknowledge a methodological limitation in our analytic strategy. Specifically, both exploratory factor analysis (EFA) and confirmatory factor analysis (CFA) were conducted on the same dataset. This approach may inflate model fit indices and does not adhere to the recommended practice of validating exploratory findings with an independent sample. Given the scale’s translation into a new language and cultural context, we initially sought to explore potential structural variations via EFA before confirming the expected six-factor structure through CFA. However, we recognize that future studies should either (a) conduct EFA and CFA on randomly split subsamples or (b) evaluate the factor structure using CFA alone, particularly when working with a well-established measure like the BMSCS. Despite this limitation, the robust psychometric findings presented here provide preliminary support for the Hebrew version of the scale and offer a foundation for further validation research.

One of the key contributions of this study lies in the linguistic and cultural adaptation of the BMSCS for use among Hebrew-speaking nurses. Validating the scale in this context ensures conceptual equivalence and measurement accuracy, supporting both clinical application and future cross-cultural comparisons. This work addresses the growing interest in self-care and mindfulness in diverse healthcare settings.

### Limitations

Firstly, although the study included a heterogeneous group of nurses, the sample sizes within specific subgroups (e.g., gender, clinical setting, or professional seniority) were not sufficiently large or balanced to support multigroup analyses. This limits our ability to assess measurement invariance, which should be addressed in future research. Additionally, since participation in this study was voluntary, nurses who completed the survey were likely to have more positive or stronger beliefs in Mindful Self-Care. It is possible that this could lead to an inflation of the outcome measure. Furthermore, the methodology cannot provide conclusive conclusions or predictive capabilities, so longitudinal studies are needed. Although the current study provides preliminary support for the psychometric properties of the Hebrew BMSCS, the sample was predominantly female. This gender imbalance precluded formal testing of measurement invariance across men and women. Given that some items (e.g., mind-body practices) may be interpreted or practiced differently based on gender, future studies with more gender-balanced samples are needed to examine whether the factor structure and item responses are invariant across gender.

## Conclusion

This study translated and evaluated the Hebrew version of the Brief Mindful Self-Care Scale (BMSCS) among Israeli nurses. The modified version of the scale demonstrated good internal consistency and an overall factorial structure that was broadly consistent with the original. However, the removal of two items to achieve model fit indicates that the original structure was not fully confirmed. These results provide initial support for the use of the BMSCS in Hebrew but underscore the importance of further validation using independent samples and culturally responsive adaptation methods.

## Supporting information

S1 Appendix Brief Mindful Self-Care Scale (BMSCS). (DOCX)
