# Supplementary material for: Psychometric properties of the Hebrew version of brief mindful self-care scale: A translation and validation study
Source: PLoS One. 2025 Aug 21;20(8):e0330524. doi: 10.1371/journal.pone.0330524 (PMC12370104; doi:10.1371/journal.pone.0330524)
Supplement: S1 Appendix — (DOCX) [file pone.0330524.s001.docx]

**Brief Mindful Self-Care Scale (MSCS)**

Check the box that reflects the frequency of your behavior (how much or how often) within the past week (7 days); never (0 days), rarely (1 day), sometimes (2 to 3 days), often (4 to 5 days), and regularly (6 to 7 days).

| **Never** | **Rarely** | **Sometimes** | **Often** | **Regularly** |
| --- | --- | --- | --- | --- |
| 0 days | 1 day | 2 to 3 days | 4 to 5 days | 6 to 7 days |
| 1 | 2 | 3 | 4 | 5 |

**Physical care**

|  | I exercised at least 30 to 60 min |
| --- | --- |
|  | I took part in sports, dance, or other scheduled physical activities (e.g., sports teams, dance classes) |
|  | I practiced yoga or another mind/body practice (e.g., Tae Kwon Do, Tai Chi) |

**Supportive relationships**

|  | I spent time with people who are good to me (e.g., support, encourage, and believe in me) |
| --- | --- |
|  | I felt supported by people in my life |
|  | I felt that I had someone who would listen to me if I became upset (e.g., friend, counselor, group) |
|  | I felt confident that people in my life would respect my choice if I said “no” |

**Mindful awareness**

|  | I had a calm awareness of my thoughts |
| --- | --- |
|  | I had a calm awareness of my feelings |
|  | I had a calm awareness of my body |

**Self-compassion and purpose**

|  | I kindly acknowledged my own challenges and difficulties |
| --- | --- |
|  | I engaged in supportive and comforting self-talk (e.g., “My effort is valuable and meaningful”) |
|  | I gave myself permission to feel my feelings (e.g., allowed myself to cry) |
|  | I experienced meaning and/or a larger purpose in my work/school life (e.g., for a cause) |

**Mindful relaxation**

|  | I did something creative to relax (e.g., drew, played instrument, wrote creatively, sang, organized) |
| --- | --- |
|  | I listened to relax (e.g., to music, a podcast, radio show, rainforest sounds) |
|  | I sought out images to relax (e.g., art, film, window shopping, nature) |
|  | I sought out smells to relax (lotions, nature, candles/incense, smells of baking) |

**Supportive structure**

|  | I kept my work/school area organized to support my work/school tasks |
| --- | --- |
|  | I maintained a manageable schedule |
|  | I maintained balance between the demands of others and what is important to me |
|  | I maintained a comforting and pleasing living environment |
